# Vitamin C as an adjuvant for treating major depressive disorder and suicidal behavior, a randomized placebo-controlled clinical trial

**DOI:** 10.1186/s13063-015-0609-1

**Published:** 2015-03-14

**Authors:** Ali Sahraian, Ahmad Ghanizadeh, Fereshteh Kazemeini

**Affiliations:** Research Center for Psychiatry and Behavioral Sciences, Department of Psychiatry, Shiraz University of Medical Sciences, School of Medicine, Shiraz, Iran; Department of Neuroscience, School of Advanced Medical Sciences and Technologies, Shiraz University of Medical Sciences, Shiraz, Iran; Shiraz University of Medical Sciences, School of Medicine, Shiraz, Iran

**Keywords:** Major depressive disorder, Ascorbic acid, Therapeutics, Clinical trial, Anti-depressive agents

## Abstract

**Background:**

There are some animal studies suggesting the possible role of vitamin C for treating depression. However, the efficacy of vitamin C for treating adult patients with major depressive disorder (MDD) has never been examined.

**Methods:**

This 8-week randomized double-blind placebo-controlled clinical trial included adult patients with major depressive disorder according to DSM-IV diagnostic criteria. Twenty-one patients in the treatment group received citalopram plus vitamin C and the 22 patients in the control group received citalopram plus placebo. The Hamilton Depression Rating Scale was used to measure depressive symptoms at baseline, week 2, week 4, and week 8. We also checked for the presence of adverse effects.

**Results:**

While depression symptoms decreased in both groups during this trial, there was no statistically significant difference between the 2 groups (*P* = .5). The rate of remission, partial response, and complete response was not different between the two groups. The rate of adverse effects were not different between the two groups.

**Conclusion:**

Adding vitamin C to citalopram did not increase the efficacy of citalopram in MDD patients. Vitamin C plus citalopram is as effective as placebo plus citalopram for treating adult patients with suicidal behavior. No serious adverse effect for this combination was identified during this trial.

**Trial registration:**

This trial was registered at http://www.irct.ir. The registration number of this trial was: IRCT201312263930N31. Date registered: 5 July 2014.

## Background

Current literature suggests the possible role of oxidative stress in the pathophysiology of major depressive disorder (MDD). While the plasma ascorbic acid level is decreased in MDD, the serum levels of superoxide dismutase (SOD) and serum malondialdehyde (MDA) as two markers of oxidation are increased [1]. Ascorbic acid prevents stress-induced cerebrocortical and hippocampal lipid peroxidation and SOD activity in mice [2]. In addition, ascorbic acid inhibits the increment of the two oxidative stress markers of glutathione reductase and glutathione peroxidase activities in a preclinical model [2]. However, a randomized, double-blind, placebo-controlled study failed to show the expected beneficial effects of vitamin C on oxidative stress in humans [3]. Clinical studies do not support that vitamin C changes the markers of oxidation in humans [4].

The water-soluble vitamin C or ascorbic acid is one of the most important antioxidants in humans [5]. Vitamin C is a part of the intracellular antioxidant system with neuroprotective effects [6].

Antidepressants are capable of reversing the changes in oxidative stress parameters in MDD patients [7,8]. A clinical trial demonstrated that the peripheral activity of the antioxidant enzyme SOD and the levels of the oxidative marker MDA were higher in MDD patients [1]. These changes were accompanied by a reduction in the levels of ascorbic acid and reverted after treatment with fluoxetine and citalopram, further reinforcing the role of ascorbic acid in MDD [1]. In addition, ascorbic acid had an effect similar to fluoxetine in an animal model of depression, reverting not only the behavioral profile induced by stress but also the oxidative damage [8].

There are some promising reports about the beneficial effect of vitamin C in animal models of depression. ‘Nevertheless, supplementary vitamins C did not decrease depression in type 2 diabetic patients in a randomized, single-blind, placebo-controlled trial’ [9].

Ascorbic acid has antidepressant-like effects in animals [8]. In addition, vitamin C potentiated the antidepressant effects of antidepressants in animals [10]. Ascorbic acid produces an antidepressant-like effect by interaction with the monoaminergic system in animal models [10]. In addition, antagonists of glutamatergic N-methyl-D-aspartate (NMDA) receptors rapidly decrease depressive symptoms [11]. Additionally, vitamin C has some anti-NMDA effects [10,12].

The results of the effects of vitamin C on depression in healthy individuals and patients with some medical conditions are contradictory. A randomized double-blind, placebo-controlled 14-day trial of sustained-release ascorbic acid including 42 healthy young adults showed that vitamin C (3,000 mg/day) increased mood measured by the Beck Depression Scale [13]. Vitamin C also decreased psychological subjective stress [14]. In a randomized double-blind placebo-controlled trial including depressed shift workers, vitamin C (500 mg/day) significantly decreased depression severity. The 21-item Beck Depression Rating Scale score decreased from 14.133 ± 4.37 to 3.233 ± 7.08 (*P* < 0.018) [15]. Nevertheless, supplementary vitamin C (1,000 mg/day) did not decrease the depression score in type 2 diabetic patients in a randomized, single-blind, placebo-controlled trial [9].

Non-controlled and limited evidence from clinical studies supports the efficacy of vitamin C for treating depressive disorder. For example, vitamin C relieved adrenocorticotropic hormone-induced depressive symptoms in a patient during a 14-day period [16]. The levels of vitamins A, C, and E in patients with depression are lower than that of the healthy controls [17]. Adding the combination of 600 mg/day of vitamin A, 1,000 mg/day of vitamin C, and 800 mg/day of vitamin E to escitalopram (10 to 20 mg/day) improved depression [17]. Nevertheless, another study reported that depression is not associated with vitamin C level [18]. The only published clinical trial that examined the efficacy of vitamin C as an adjuvant agent for treating MDD included pediatric patients. There were 12 patients in the fluoxetine (10 to 20 mg/day) plus vitamin C (1,000 mg/day) group. The control group included 12 patients administered fluoxetine (10 to 20 mg/day) plus placebo. Depression score decreased in the treatment group more than that of the control group during 3 months as measured by the Children’s Depression Rating Scale (CDRS) [19]. However, the scale of Clinical Global Impression (CGI) did not show the efficacy of vitamin C for treating patients with MDD in the same trial [19]. Furthermore, suicidal behavior is common in patients with MDD and oxidative stress and lower total antioxidant levels are associated with a history of suicidal attempts [19].

Considering the preliminary reports from animal studies, a case report about the efficacy of vitamin C [16], the contradictory results of one clinical trial on adolescents with depression [19], and the role of oxidative stress in MDD and suicidal behavior, well-controlled trials are needed to examine whether the effect of vitamin C as an adjuvant medication improves MDD in adults. To the best of the authors’ knowledge, this matter has never been examined in adult patients with MDD. Therefore, we conducted a randomized double-blind placebo-controlled clinical trial to examine the efficacy and safety of vitamin C for treating MDD and suicidal behavior. We hypothesized that vitamin C would augment the antidepressant effects of citalopram in treating adult patients with MDD.

## Methods

The study was performed in accordance with the Declaration of Helsinki (1964). This trial was approved by the Ethics Committee of Shiraz University of Medical Sciences (number: CT-P-92-6070). The participants were provided with information about the trial procedure, risks, and benefits. Then, the participants provided their written informed consent. This trial is registered in the IRCT (IRCT201312263930N31). This trial was performed at the Department of Psychiatry at Shiraz University of Medical Sciences from September 2013 to March 2014.

### Participants

This is a randomized controlled clinical trial investigating the effectiveness of vitamin C in addition to citalopram for treating MDD. Participants were 60 adult outpatients who met the *Diagnostic and Statistical Manual of Mental Disorders, fourth edition* (DSM-IV) criteria for MDD. Diagnosis was made through a clinical interview by a board-certified psychiatrist. There were two groups. One group received citalopram (up to 60 mg/day) plus vitamin C (up to 1,000 mg/day). The other group received citalopram plus placebo. Citalopram was started with 10 mg/day and increased to 20 mg/day over 7 days. The dosage could be adjusted according to adverse effects. No other antidepressant medication or psychotherapy was administered during this trial. The patients and/or caregivers were asked whether they took the medications in order to check the patients’ medication compliance. Tablets of vitamin C and placebo were identical. Placebo tablets were made by the Faculty of Shiraz University of Medical Sciences. The patients, the rater, and the clinician who referred the patients were blind to the group allocation.

The inclusion criteria were: a score of 16 or higher on the Hamilton Depression Rating Scale (HDRS); no psychotropic medication for at least 4 weeks; untreated patients; both genders; and aged more than 18 years and no more than 65 years old.

The exclusion criteria were: 1) significant co-morbid psychiatric illness, hypothyroidism or severe medical conditions such as epilepsy; 2) psychotic features or bipolar mood disorder or any other psychiatric disorder; current substance abuse except cigarette smoking; suicidal attempt; oral contraception use; pregnancy; breast-feeding; intention to become pregnant during this trial; a history of allergic reactions to vitamin C or citalopram; and receiving psychotropic medication. Suicidal ideation was not an exclusion criterion.

### Outcomes

The patients were assessed 4 times including at baseline, week 2, week 4, and week 8. The HDRS-21 was the primary outcome measure to assess depression severity [20]. The item 3 was scored to measure suicidal behavior. Mean difference of HDRS was compared between the two groups. Reduction of more than 49% in score from baseline to week 8 was defined as response [21]. The 2 groups were compared regarding complete response (# 50%), partial response (# 25% decline in HDRS score), and the rate of adverse effects. The rate of full remission (HDRS less than 8) was compared between the 2 groups [22]. The secondary outcome measure was the Beck Depression Inventory (BDI).

### Adverse effects

A checklist was used to check adverse effects. In addition, the patients and caregivers were requested to report any adverse effects. They were provided with a phone number to contact us whenever any adverse effect or any question about the trial arose.

### The procedure

The order of entrance in the trial was used for randomization. A random number generator provided a list. The rater and the man who randomized the patients into the groups were two different persons.

#### Sample size

A published clinical trial showed that adding vitamin C to fluoxetine therapy in 12 children with MDD decreased the CDRS score. This scale is derived from the HDRS. The score decreased from 30.1 ± 3.56 to 14.4 ± 2.39. Meanwhile, the score in the placebo group decreased from 31.3 ± 3.89 to 24.7 ± 2.73 [19]. To determine the sample size, the result of the previous study was used [19]. A total of eight patients are needed to enter this two-treatment parallel-design study. The probability is 98% that the study will detect a treatment difference (*P*-value < 0.05 at a 2-sided, df = 9.0, SD = 2.5).

### Statistical analysis

SPSS software (Chicago, IL, USA) was used to run the statistical analysis. Continuous variables were compared between the two groups using two-sample *t*-tests to examine differences in means. Repeated measures analysis of variance (ANOVA) was used to compare the scores between the two groups. Group was considered as a between subjects factor and the four measurements during trial (time) were considered as a within subject factor. Repose rates, remission rate, and categorical variables were compared between the two groups using Chi square test or Fisher’s exact test. The last observation carried forward method was used for handling missing data. The patients who were assessed at least twice were analyzed. A *P* -value less than 0.05 was set as significant level.

## Results

**Figure 1:**
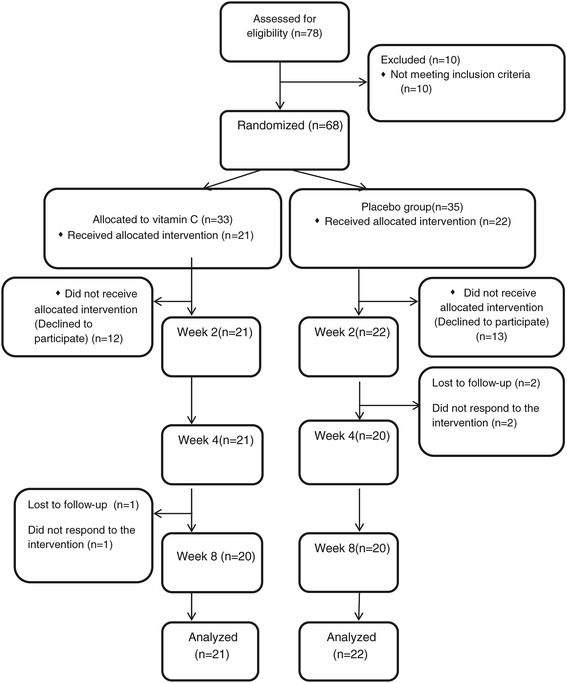
**Consolidated Standards of Reporting Trials (CONSORT) flowchart of the patients in this trial.**

The flow of patients in the trial is displayed in Figure 1. Seventy-eight patients were screened to enter this trial. Ten patients were excluded because the severity of depression according to HDRS was not severe enough. From the 68 patients who were randomly allocated into one of the two groups, 35 patients were in the placebo group and 33 patents were allocated into the vitamin C group. Twelve patients in the vitamin C group and 13 patients in the placebo group declined to participate. From 22 patients in the vitamin C group, 1 patient dropped out due to lack of efficacy of the intervention. Two patients in the placebo group dropped out because the intervention was ineffective. All 43 patients who did not decline to participate had at least 1 follow-up assessment. In all, 40 patients completed this trial. These 43 patients were included in this intent-to-treat analysis.

The mean age of the patients who declined to participate in this trial was not different from those who participated (31.0 (9.6) versus 33.5 (9.4) years old, respectively; t = 1.02, df = 65, *P* = .3). Moreover, the HDRS score at baseline was not different between the patients who declined to participate and those who participated (23.2 (4.9) versus 22.2 (3.2); respectively; t = 1.06, df = 66, *P* = .2). In addition, their gender ratio was not different (X2 = .8, df = 1, *P* = .3).

The rate of females in the vitamin C and placebo groups was 15 (71.4%) and 17 (77.2%), respectively (X2 = .19, df = 1, *P* = .6). The age range of the patients was from 19 to 55 years old. The mean age of patients in the vitamin C and placebo group was 32.7 (8.4) and 34.2 (10.3) years old. There was no statistically significant difference between the 2 groups regarding the mean of age (t = 1.5, df = 41, *P* = .5).

None of the patients had any psychotic features (Table 1). None of the patients had a positive history of being administered electroconvulsive therapy. Body Mass Index of the patients and the mean dosage of citalopram were not different between the two groups.

**Table 1:** **The characteristics of the patients and depression score and its changes in the groups**

		**Vitamin C group (n = 21)**	**Placebo group (n = 22)**	
Mean (SD) years of age		32.7 (8.4)	34.2 (10.3)	t = 1.5, df = 41, *P* = .5
Number (%) of females		15 (71.4)	17 (77.2%)	X2 = .19, df = 1, *P* = .6
Marital status n (%)	Single	7 (31.9%)	7 (31.8%)	X2 = .1, df = 2, *P* = .9
	Married	13 (59.1%)	14 (63.6%)	
	Divorced	1 (4.5%)	1 (4.5%)	
Psychotic features		0	0	-
Number (%) of smokers		2 (9.1%)	2 (9.1%)	-
Positive history of being administered ECT		0	0	-
Mean (SD) of Body Mass Index		25.1 (3.4)	26.3 (6.5)	t = .7, df = 42, *P* = .4
Citalopram mean (SD) dosage (mg/day)	During first 2 weeks	20.2 (0.)	20.9 (4.2)	t = −1.0, df = 42, *P* = .3
	During weeks 3 and 4	24.0 (6.6)	24.5 (7.3)	t = −.2, df = 42, *P* = .8
	During second month	35.0 (7.4)	33.5 (7.4)	t = −.6, df = 40, *P* = .5
Hamilton Depression Rating Scale score	Baseline	21.4 (4.1)	22.4 (3.0)	t = −.9, df = 42, *P* = .3
	Week 2	15.0 (5.6)	16.9 (6.0)	t = 1.0, df = 42, *P* = .4
	Week 4	11.2 (6.8)	11.9 (5.9)	t = −.3, df = 41, *P* = .7
	Week 8	8.6 (5.2)	9.3 (6.3)	t = −.3, df = 41, *P* = .6
Decline of Hamilton Depression Rating Scale score (%)		60.0 (20.3	59.2 (26.9)	t = −.1, df = 41, *P* = .9
Mean (SD) of Beck Depression Inventory score	Baseline	23.9 (8.3)	22.8 (9.8)	t = −.3, df = 41, *P* = .6
	Week 2	18.0 (11.1)	17.9 (9.8)	t = .02, df = 41, *P* = .9
	Week 4	14.8 (10.8)	15.1 (10.4)	t = .1, df = 41, *P* = .9
	Week 8	8.6 (10.7)	9.0 (9.6)	t = −.1, df = 41, *P* = .9
Mean (SD) decline of suicidal score		1.0 (0.9)	0.9 (0.9)	t = −.3, df = 37, *P* = .7

*Abbreviation*: *ECT* electroconvulsive therapy.

### Efficacy

**Figure 2:**
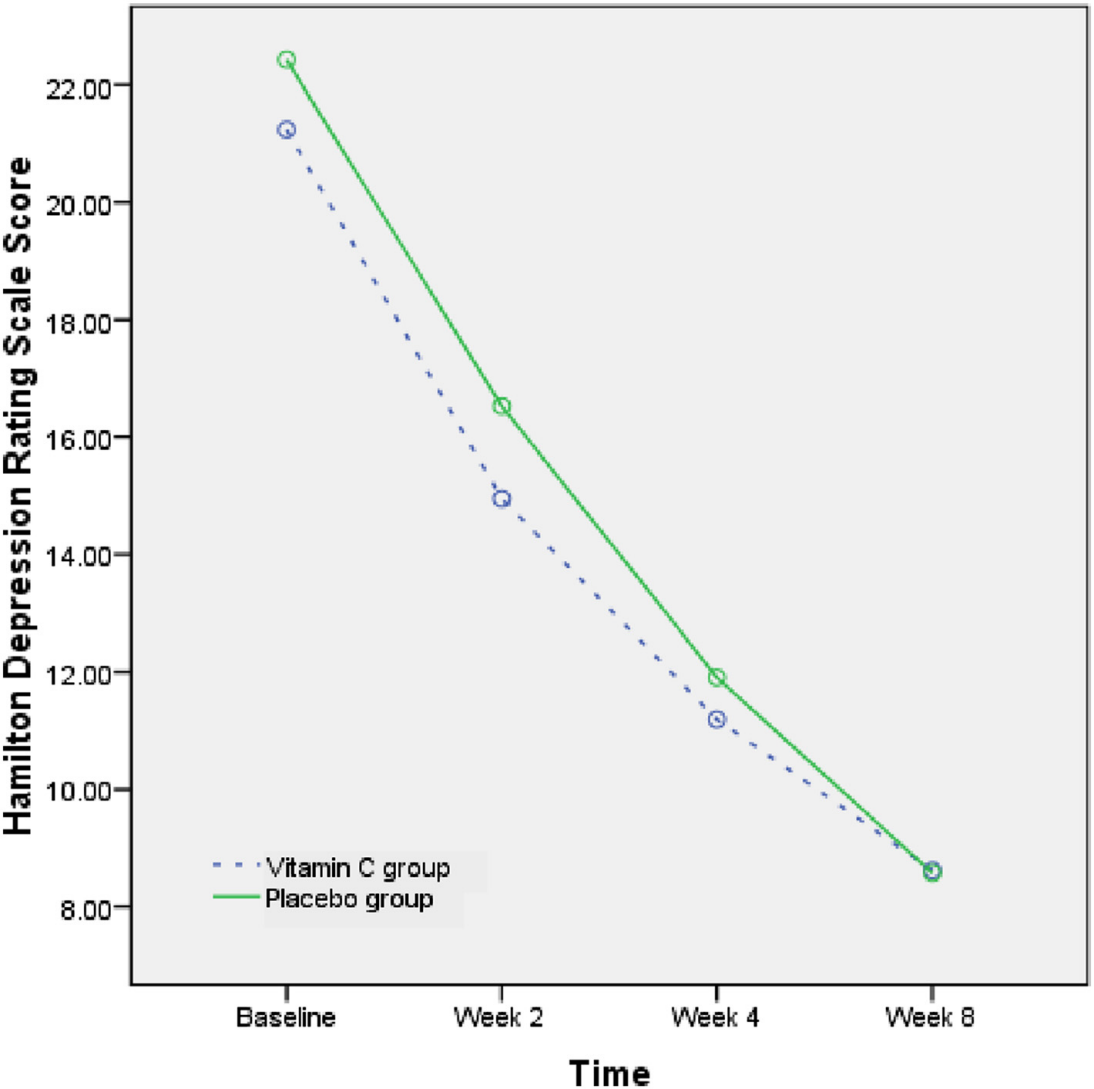
**The mean change of Hamilton Depression Rating Scale in the groups during the trials.**

The mean (SD) scores of HDRS are displayed in Table 1. The HDRS score was not different between the two groups at baseline. Repeated measure of ANOVA showed that the HDRS score significantly decreased during this trial in both groups (F(3,120) = 154.6, *P* < 0.001) (Figure 2). However, the interaction of time and group (time × group) was not statistically different (F(3,120) = .57, *P* = .6). In other words, the behavior of the two treatments was similar across time. In addition, repeated measure ANOVA showed that there was no statistical difference between the 2 groups (F(1,40) = .33, *P* = .5). Additionally, the difference between the 2 groups was similar at the endpoint (t = −.3, df = 41, *P* = .6).

The percent of decline of HDRS in the vitamin C and placebo groups was 60.0% and 59.2%, respectively (t = −.1, df = 41, *P* = .9).

### Remission rate

The rate of remission was not different between the 2 groups (7 (35.0%) versus 8 (36.4%); X2 = 0.008, df = 1, *P* = .4).

### Complete response rate

Sixteen (48.5%) of the patients in the vitamin C group and 17 (51.5%) of the patients in the placebo group showed a complete response (X2 = 0.04, df = 1, *P* = .8).

### Partial response rate

The rate of partial response was not different between the two groups (20 (100.0%) versus 19 (86.4%); X2 = 2.9, df = 1, *P* = .08).

### Suicidal behavior

The mean score of suicidal behavior in the vitamin C and placebo groups at baseline was 1.7 (1.4) and 1.7 (1.3), respectively. It was not different between the two groups (t = −.02, df = 41, *P* = .9). The suicidal score decline in the vitamin C and placebo group was 1.0 (0.9) and 0.9 (0.9), respectively.

### Beck Depression Inventory

The mean (SD) score of the BDI was not different between the two groups at baseline (Table 1). The BDI score decreased from 23.9 (8.3) to 8.6 (10.7) in the vitamin C group. The score decreased from 22.8 (9.8) to 9.0 (9.6) in the placebo group.

### Clinical adverse effects

There was no serious adverse effect. Nobody dropped out due to an adverse effect. Three patients stopped the interventions because it was ineffective.

None of the adverse effects were so severe to necessitate a change of intervention protocol. The adverse effects were transient and resolved spontaneously. The most common adverse effects in both groups were nausea, dry mouth, pain, sweating, and decreased appetite. Table 2 shows that the rates of adverse effects were not different between the two groups.

**Table 2:** **The rate of adverse effects by group**

**Adverse effect**	**Vitamin C group n (%)**	**Placebo group n (%)**	
Nausea	12 (57.1)	11 (52.4)	X2 = 0.09, df = 1, *P* = .7
Fatigue	9 (42.8)	8 (38.1)	X2 = 0.09, df = 1, *P* = .5
Dry mouth	7 (33.3)	9 (42.9)	X2 = 0.4, df = 1, *P* = .5
Pain	6 (28.6)	8 (38.1)	X2 = 0.4, df = 1, *P* = .5
Sweating	0	4	-
Decreased appetite	4 (19)	6 (28.6)	-
Abdominal pain	3 (14.3)	3 (14.3)	-
Increased appetite	3 (14.3)	0 (0.0)	-
Restlessness	3 (14.3)	2 (9.5)	-
Insomnia	3 (14.3)	1 (4.8)	-
Blurred vision	0	3 (14.3)	-
Itch	0	2 (9.5)	-
Tremor	0	2 (9.5)	-
Dizziness	1 (4.8)	2 (9.5)	-
Daytime drowsiness	2 (9.5)	0	-
Drooling	1 (4.8)	0 (0.0)	-
Nervousness	1 (4.8)	1 (4.8)	-
Constipation	0	1 (4.8)	-
Lethargy	0	1 (4.8)	-
Tachycardia	0	1 (4.8)	-
Dystonia	0	0	-
Diarrhea	0	1 (4.8)	-

## Discussion

The results of this randomized double-blind placebo-controlled clinical trial indicate that the administration of vitamin C as an adjuvant medication with citalopram did not increase the efficacy of citalopram for treating depressive symptoms in patients with MDD. The rates of partial response, complete response, and remission were not different between the two groups. Vitamin C as much as placebo decreased depression symptoms. These findings are not in line with the results reported from animal studies [8,12,23,24]. Nevertheless, this is the first randomized controlled clinical trial examining the effectiveness and safety of vitamin C for treating adult patients with MDD. It should be noted that there is a difference between animal studies and major depression in humans. The animal studies measured depression induced by stress. It is clear that the stress-induced response is not equal to MDD.

The only trial conducted in humans reported contradictory results about the effectiveness of vitamin C for treating depression symptoms in adolescents [19]. That trial reported that vitamin C did not decrease depression symptoms measured by the CGI scale. However, the depression symptoms were measured by the CDRS [19]. In addition, the other trial which showed the efficacy of vitamin C had been conducted on healthy individuals [13] rather than patients with MDD.

Considering that oxidative stress is associated with depression [25], it was expected that vitamin C would decrease depression symptoms and suicidal behavior. Individuals with depression markedly intake dietary antioxidants less than those without depression [26]. Vitamin C has a protective role for oxidative status in stress [2]. In addition, some anti-inflammatory agents such as celecoxib effectively decrease inflammatory markers and depression symptoms in patients with MDD [27,28]. Nevertheless, our results failed to support this assumption. This is in line with a study that vitamin C level was not associated with depression symptoms in community-based adults [29]. Another explanation is that although MDD is associated with oxidative stress the effect of antidepressants is independent of oxidative and antioxidative stress systems [25].

The ceiling effect may be another explanation for the negative results of this trial. It means that citalopram has masked the potential benefit of vitamin C. The animal studies did not concurrently administered citalopram. To eliminate the ceiling effect, a monotherapy trial of vitamin C compared to placebo is needed.

Suicidal behavior and serotonin-specific reuptake inhibitor-related emergent suicidal ideation and behavior are a matter of clinician’s concern in treating patients with MDD. However, efficacy results for this trial showed that vitamin C was not statistically different from placebo for treating patients’ suicidal ideation and behavior. Regarding the adverse effects, the rates of adverse effects were not different between the two groups.

While no double-blind randomized controlled clinical trial has ever examined the effect of vitamin C for treating depression symptoms in adults patients with MDD, the small sample size, short duration, and fixed dose of vitamin C were among the limitations of this trial. The current trial included patients without psychosis. It is not clear whether vitamin C is effective for treating patients with severe forms of MDD or those with psychotic features. Although some studies have reported that the level of vitamin C is not associated with depression symptoms, the current trial did not control the dietary intake of vitamin C. Moreover, it is possible that the dose of vitamin C was insufficient. Although none of the patients in the current trial were psychotic and the severity of depression at baseline was not different between the two groups, future studies should consider duration and age of onset of depression as possible co-variate factors. Finally, 25 out of 68 patients declined to participate in this trial; they may be a source of potential bias.

## Conclusion

Treating MDD with vitamin C adds nothing to the short-term efficacy of citalopram. This combination is not effective regarding suicidal behavior. However, this combination seems to be safe and well-tolerated.

## Abbreviations

MDD: Major depressive disorder
SOD: superoxide dismutase
MDA: serum malondialdehyde
NMDA: N-methyl-D-aspartate
CDRS: Children’s Depression Rating Scale
HDRS: Hamilton Depression Rating Scale
